# Psychological therapies for bipolar disorder in later life: Current evidence, practice and future directions

**DOI:** 10.1111/papt.12593

**Published:** 2025-04-04

**Authors:** Elizabeth Tyler, Aaron Warner

**Affiliations:** ^1^ Division of Psychology and Mental Health University of Manchester Manchester UK; ^2^ Department of Primary Care & Mental Health University of Liverpool Liverpool UK

**Keywords:** bipolar disorder, older adults, psychological therapies, randomised controlled trial, recovery, therapy adaptations

## Abstract

**Purpose:**

Bipolar disorder (BD) is a severe and enduring mental health condition that persists into older adulthood. The number of people living with BD into later life is set to increase as our population ages and awareness of the condition increases. BD in later life may present with additional challenges, such as increased physical health comorbidities and poorer cognitive function. Despite these additional challenges, there has been a paucity of research focused specifically on the treatment needs of older people with BD, highlighted by several review papers. In comparison, the last 30 years have witnessed a rapid development of psychological treatments for younger people with BD.

**Methods:**

The authors completed a literature review of peer‐reviewed journal articles reporting on psychological therapies developed specifically for older adults with BD. The authors also considered adaptations to psychological therapies that may benefit this population.

**Results:**

This review highlighted that there is limited available research investigating the efficacy of psychological treatments for older adults with BD, despite their unique care needs. The available evidence demonstrated the importance of adapting psychological interventions to meet this client group's needs and outlined potentially beneficial adaptations, such as increasing the flexibility of treatment, taking into account health‐related and symptom changes, and developing treatments in consultation with older adults with BD.

**Conclusions:**

Improved attention and awareness of the challenges faced by older adults with BD is warranted. The development of tailored psychological treatments for this group may help reduce the significant inequalities they currenty face.

## INTRODUCTION

Bipolar disorder (BD) is a mental health condition that is characterized by severe fluctuations in mood (mania, hypomania and depression) (American Psychiatric Association, 2013). The difficulties associated with BD often lead to diminished well‐being, poor quality of life, and high rates of suicidality (Grande et al., 2016). BD affects approximatey 1% of the world's population (Grande et al., 2016). As the population continues to age, the number of people living with BD in later life is expected to rise dramatically (Depp & Jeste, 2004). This is problematic, as older adults experiencing severe mental health difficulties such as BD face different challenges from younger age groups, such as higher rates of physical health comorbidities and cognitive decline (Bahorik et al., 2017; Gildengers et al., 2009; Warner et al., 2023). Despite these concerns, the literature surrounding older adults with BD remains sparse, and interventions continue to focus predominantly on the needs of younger adults with BD (Sajatovic et al., 2015). Consequently, older adults with BD require improved support to address the health inequalities they currently experience (Sajatovic et al., 2015).

The National Institute for Health and Care Excellence (NICE, 2014) currently recommends that older adults with BD should be offered the same psychological treatments as younger adults. Yet, existing literature identifies that older adults with BD experience unique, complex challenges that potentially require adaptations for treatments to be effective (Warner et al., 2024). A systematic review by Warner et al. (2023) indicated that older adults with BD experience a high prevalence of physical health comorbidities such as cardiovascular disease. Research also highlights that this group experiences prolonged episodes of depression and mania and shorter intervals between episodes (Nivoli et al., 2014). Poorer cognitive function and a higher prevalence of psychiatric comorbidities such as panic disorder, alcohol use disorder, and generalized anxiety disorder have also been reported when compared to age‐matched controls without BD (Goldstein et al., 2006; Schouws et al., 2009). These challenges may also be exacerbated by ageing related social changes such as retirement, caregiving, care receiving, and financial difficulties that may negatively impact the quality of life of older adults with BD (Dave et al., 2008; Dening & Barapatre, 2004). This evidence suggests that increased attention is required to support this group (Sajatovic et al., 2015). Despite this, literature considering what adaptations are required to improve support for older adults with BD remains sparse (Tyler et al., 2021).

Treatments for older adults with BD typically involve the long‐term use of mood‐stabilising medication (Morlet et al., 2019). Whilst these treatments can be effective for many, research investigating their impact over the long term is limited (Warner et al., 2023). Some evidence suggests that the long‐term use of medications such as lithium can decrease the risk of Alzheimer's (Nunes et al., 2007). However, the long‐term use of mood‐stabilising medication has also been linked to weight gain, cognitive challenges, and diabetes, which may have significant consequences for individuals with BD in later life (Lala & Sajatovic, 2012). Recent research has suggested that older adults with BD value treatments that enable them to build upon age‐related strengths, feel part of the community, support others, and work towards their personal goals rather than prioritising symptom reduction alone (Tyler et al., 2022; Warner et al., 2024). These findings suggest that psychological interventions may be beneficial for this group, although adaptations are required to support older adults with BD to meet these goals.

This article will identify and summarise the current evidence base for psychological therapies developed specifically for older adults with BD. The authors will also consider what adaptations have been used to develop psychological therapies for older adults with BD and identify future directions that may help to improve support and quality of life among this group. In this review, older adults are defined as aged 60 and over, as this is consistent with the World Health Organisation's chronological definition of elderly, aged, or older adults (World Health Organisation, 2021).

## CURRENT PUBLISHED AND ONGOING INTERVENTIONS FOR OLDER PEOPLE WITH BD

A systematic search of randomised controlled trials of psychological interventions developed specifically for older people with BD was conducted in May 2024. The initial inclusion criteria for the studies specified that the sample consisted solely of individuals with a diagnosis of BD, participants aged 60 and older, a psychological intervention, and a randomised controlled trial. Studies were identified by systematically searching the following four electronic databases: Web of Science, Pubmed, Embase, Medline, Central (COCHRANE). Search strings for BD AND older adults AND psychological interventions AND randomised controlled trials were used. A total of 156 articles were screened at the title and abstract stage. Fourteen were included for a full paper review. One RCT of a psychological intervention developed specifically for older people with BD was found (Tyler et al., 2022).

A systematic search of the ISRCTN Registry, ClinicalTrials.gov and the World Health Organisation trial databases was performed using search terms ‘bipolar’, ‘older’ and ‘therapy’ to identify any completed or ongoing clinical trials of psychological interventions for older people with BD. Tyler et al.'s (2022) completed trial was retrieved from the ISRCTN registry and the World Health Organisation trial database. Only one ongoing RCT evaluating the efficacy of a group‐based functional remediation program for older adults with BD was found and a protocol paper was retrieved from the ClinicalTrials.gov database (Montejo et al., 2022).

Due to the lack of retrieved studies, the search was broadened, and two additional studies were identified via Google Scholar searches, cited articles, and reference list checks (Burgin & Gibbons, 2016; Depp et al., 2007).

The final result of these searches identified four published studies on psychological interventions developed specifically for older people with BD. These are as follows:

- Medication adherence skills training intervention (Depp et al., 2007).
- Narrative therapy (single case; Burgin & Gibbons, 2016)
- Recovery‐focused therapy (Tyler et al., 2016, 2022).
- Functional Remediation (Protocol paper; Montejo et al., 2022).

## MEDICATION ADHERENCE TRAINING

### Focus of the intervention

Depp et al. (2007) developed a medication adherence skills training intervention for older adults with BD. They focused on medication adherence as the primary outcome due to its common and modifiable risk for poor outcomes across the lifespan (Greil & Kleindienst, 2003), especially for older adults with BD who face practical challenges such as changes in cognition and medications for multiple comorbidities. These factors impact the ability to maintain medication adherence in later life, which is not addressed in existing psychotherapy protocols for younger people with BD.

Depp et al. (2007) highlighted that medication non‐adherence can result from intentional (e.g. negative attitudes towards taking medication) and unintentional factors such as forgetting. Additionally, older adults, who often have multiple medications due to various medical conditions, are at an increased risk of side effects (Sajatovic, 2002; Vik et al., 2004).

Depp et al. (2007) designed a 12‐week group intervention to address medication knowledge, attitudes, and management skills. The multi‐component intervention included education, motivational training, medication management (to reduce intentional and unintentional adherence) and developing cognitive and behavioural skills to reduce the impact of depression symptoms.

### Results

Twenty‐one older adult outpatients with BD participated in this study (mean age 60  years, SD = 6; age range 53–73 years). At baseline, 55% of participants were not adherent to psychiatric medication for BD. On average, these participants experienced moderately severe depression and minimal symptoms of mania. Seventy‐six percent of participants completed the intervention. Results suggested that medication adherence skills training was a feasible, acceptable intervention for older adults with BD (Depp et al., 2007). Among participants who completed the intervention, the percentage of participants reporting non‐adherence to medication reduced by 15% and the percentage of participants reporting difficulties taking medication reduced by 31%. Small to medium effect sizes in pre‐post improvements in medication adherence, depressive symptoms, medication management ability, and selected domains of health‐related quality of life were observed by Depp et al. (2007).

The high retention rates reported by Depp et al. (2007) suggest that older adults with BD may be motivated to engage in psychosocial treatments aiming to reduce barriers to medication adherence. Whilst these results are promising, the authors note that findings are based on a small sample size, making it difficult to determine which factors predicted treatment effectiveness. Furthermore, there was no control condition or follow‐up data to assess the long‐term impact of the intervention. Despite these limitations, the study offers insight into the benefits of psychosocial interventions alongside medication use for older adults living with BD.

## RECOVERY‐FOCUSED THERAPY

### Focus of the intervention

Tyler et al. (2022) developed a recovery‐focused psychological therapy for older people with BD, based on Jones et al.'s (2015) therapy for a younger cohort. Interventions for younger cohorts have mainly used CBT techniques to target clinical improvement (e.g. reducing relapse and frequency/severity of mood episodes). However, this has been deemed impossible for some (Leonhardt et al., 2017) and the personal recovery movement, led by service users, has called for a new approach to define ‘recovery’. This has led to a shift from recovery outcomes based on symptom eradication and ‘cure’ to a focus on building existing strengths and resilience.

CBT traditionally views the person as ‘vulnerable’ and focuses on overcoming deficits. RfT (Jones et al., 2015) takes a different approach, emphasising pre‐existing strengths and client‐orientated goals (such as relationships, social engagement, or work/ voluntary work) rather than a presumption that the primary target for therapy is reducing symptoms and clinical relapse.

The RfT approach was considered highly appropriate for working with older people with BD for several reasons. It aligns with service user priorities, focusing on areas of personal growth such as building assertiveness and enhancing confidence and competence (Tyler et al., 2021). It promotes a collaborative approach, empowering individuals to take control of their lives. The approach offers flexibility to address various problems based on individualised needs. The RfT strengths‐based approach is also consistent with Kadri et al.'s (2022) intervention where they used a wisdom‐enhancement timeline technique to target symptoms of depression in older people.

Tyler et al. (2022) adapted the original RfT manual to meet the needs of an older adult population. This included extensive consultation (focus groups and one‐to‐one) with service users with lived experience of BD in later life, their relatives, and experts in the field (see Tyler et al., 2021).

### Results

Tyler et al. (2022) recruited 39 older adults with BD (mean age 67 years, SD = 6; range 60–81 years) to take part in a parallel, two‐armed randomised controlled trial comparing RfT for older people (fT‐OA) to treatment as usual. Findings from the study suggested that RfT‐OA was an acceptable and feasible intervention for older adults with BD. Participants included in the intervention arm of the trial demonstrated a significant commitment to engaging in RfT‐OA. Tyler et al. (2022) also completed qualitative interviews as part of the study. These interviews indicated that participants valued RfT‐OA and highlighted its positive impact on their relationships with family, work colleagues, and lifestyle. The recovery approach, which prioritised participants' strengths and enabled them to work towards personal values‐driven goals, was deemed beneficial by participants. Although this study included a small sample consisting of predominantly white‐British individuals, it highlighted that older people with BD value psychological interventions.

## NARRATIVE THERAPY

### Focus of the intervention

In their 2016 study, Burgin and Gibbons detailed the use of narrative therapy with an older individual diagnosed with BD (Burgin & Gibbons, 2016). Narrative therapy involves assisting people in creating new, more helpful personal stories by identifying their values and skills history (Brown, 2007). Burgin and Gibbons (2016) highlighted that narrative therapy can be used with a broad range of clients and does not focus on specific problems. They also discussed the challenges faced by older individuals, such as changes in functioning due to the loss of a partner, changes in self‐care ability following illness or injury, and shifts in social experiences during retirement or changes in sexual relationships (Kropf & Tandy, 1998).

### Results

This article presented a case illustration of a 61‐year‐old Caucasian client diagnosed with BD aged 21. Burgin and Gibbons (2016) reported that narrative therapy helped the client plan the rest of her life, establish a renewed vision, and find a greater sense of meaning in her life. By reconstructing her life story, the client formulated a newfound identity that enriched her later years as a mother, grandmother, and artist. Narrative therapy's focus on strengths rather than difficulties associated with BD was critical to this significant shift. This focus potentially helped empower the client to reconcile past challenges and discover new hobbies, enabling her to live well with BD in later life (Kenyon & Randall, 1999).

The authors reported several limitations when using the narrative therapy approach. While narrative therapy allows clinicians to develop an in‐depth understanding of clients' difficulties across their lifespan, this can take several sessions. Some older adults with BD continue to experience fluctuations in mood, which may disrupt the flow of sessions and the content explored. When using narrative therapy, clients must also complete tasks outside therapeutic encounters, such as reflective writing (Kennedy, 2008). To fully engage, clients must be highly committed to therapy, which may be overwhelming (De Vries et al., 1990). Despite these limitations, Burgin and Gibbons (2016) demonstrate that narrative therapy can be a valuable therapeutic model to support older adults in making sense of challenges associated with BD across their lifespan, instil hope, and empower them to live fulfilling lives.

## FUNCTIONAL REMEDIATION

### Focus of the intervention

Montejo et al. (2022) have adapted a functional remediation program for an older population (FROA‐BD) to address common functional, cognitive, and physical health challenges in older adults with BD (aged over 60). Their protocol emphasises targeting cognitive performance as an outcome in therapy, given that ~50% of older people with BD present with cognitive impairment (Gildengers et al., 2004).

FROA‐BD is based on the Functional Remediation Program (Torrent et al., 2013), which consisted of 90‐min weekly sessions over 21 weeks. The sessions included psychoeducation about cognitive deficits, developing coping strategies, improving communication and autonomy, and managing stressful situations.

The FROA‐OA program has been adapted for older people by increasing the number of sessions to twice a week over a 4‐month period (*N* = 32). The content has been tailored to address topics more relevant to the targeted age group, such as sessions on language, visuospatial ability, physical exercise, and diet recommendations. There are additional sessions for relatives to explain how cognition can be affected in later life by BD and the role of family and caregivers.

The authors suggest that the results of the upcoming trial will provide important evidence of how functional remediation can improve cognitive performance and psychosocial functioning among older adults with BD. This study may have vital implications for the development of psychological treatments for older adults with BD, although it is currently at the protocol stage.

## HOW HAS THERAPY BEEN ADAPTED FOR OLDER PEOPLE WITH BD?

### Guidelines for adapting therapy for older people

Both Tyler et al. (2022) and Depp et al. (2007) used guidelines developed for adapting therapy for older people in general to adapt their psychological interventions for later life BD. Depp et al. (2007) used Areán et al.'s (2003) ‘Guidelines for Conducting Geropsychotherapy Research’. A summary of their guidelines focused on making age‐ appropriate adaptations to therapy for older adults in general is detailed in Table 1 below.

**TABLE 1:** Summary of Areán et al.'s (2003) guidelines for age‐appropriate adaptations to therapy for older adults.

Patient education	Initial sessions must include socialisation to the therapy to address any misconceptions (Areán et al., 2003) Provide written information about therapy (Areán et al., 2003)
Adjusting for changes in information processing	Decrease the session length from the standard 50–30 min enabling clients to maintain therapeutic focus (Areán et al., 2003) Use ‘cue and review’ (Alexopoulos & PROSPECT Group, 2001) ○ Involves the therapist working systematically through each step of a problem‐solving skill and reviewing what the client has achieved ○ The entire process is then reviewed at the end of the session
Increasing the flexibility of treatment to accommodate social and functional constraints	Ensure strategies used in therapy match the developmental process of the older person (Areán et al., 2003) ○ Encouraging older people to engage in activities that are opposed to the developmental trajectory can result in less benefit than good (Antonucci & Akiyama, 1987) Research on social adaption indicates as a person ages, they become more selective about who they socialise with and may have smaller social networks (Isaacowitz & Seligman, 2001) ○ Therefore, in CBT, encouraging older people to expand their social networks to enhance their mood may not always be appropriate
Increasing the flexibility of treatment to accommodate social and functional constraints	Psychological therapy must be flexible in structure, location and the presentation of treatment (Areán et al., 2003) ○ Use forms with larger prints ○ Audio‐taping sessions and details of homework ○ Consider offering to deliver the therapy in non‐mental health settings such as the client's home, churches and day centres

Tyler et al. (2022) used Chand and Grossberg's (2013) recommendations for adapting cognitive behavioural therapy for older adults in general. These adaptations are summarised in Table 2 below.

**TABLE 2:** Summary of Chand and Grossberg's (2013) recommendations for adapting cognitive behavioural therapy for older adults.

Loss and transitions	CBT for older adults should focus on the meaning a client places on losses and transitions in later life (Chand & Grossberg, 2013) ○ For example. a depressed person may view their retirement from work and becoming less productive as a ‘loss of self‐worth’ ○ CBT in later life can help clients identify new ways of thinking about situations which can help them adapt to their losses and transitions
Changes in cognition	Chand and Grossberg (2013) present strategies to adapt to older adults' changes in cognition in later life. These include: Information presented slowly Frequent repetition and summaries Encouraging clients to take notes during sessions Present new information in the context of previous experiences to promote learning Making recordings of session which clients can listen to in‐between sessions Using phone prompts and alarms to remind clients to carry out therapeutic tasks such as breathing exercises in‐between sessions Involving a trusted other in sessions to act as a co‐therapist at home (only with consent from both parties)

### Clinical frameworks for working with older people

Tyler et al. (2022) used Knight and Poon's (2008) Contextual Adults Life Span Theory for Adapting Psychotherapy with Older Adults (CALTAP) and Laidlaw et al.'s (2004) comprehensive conceptualisation of CBT which offer a framework to help guide clinicians when working therapeutically with an ageing population in general. Both models take similar themes into account such as the impact of environmental factors (e.g. cohort effects) and age‐related challenges (e.g. changes in physical health). Additionally, the impact of losses and transitions is emphasised by both models. Laidlaw et al.'s (2004) case conceptualisation builds upon Beck's (1979) standard CBT model to include additional information which may be important when working with older people. These are summarised below in Table 3.

**TABLE 3:** Summary of Laidlaw et al.'s (2004) comprehensive conceptualisation of CBT for working therapeutically with an ageing population.

Cohort beliefs	Cohort beliefs are the beliefs held by a group of people born during the same time period Each era involves historical, social, and cultural influences that impact a person's life and development Understanding cohort beliefs and core beliefs is important for providing age and generational context in therapy (Evans, 2007)
Role investments	Later in life, there may be changes and transitions in roles Role investment refers to staying actively involved in meaningful activities and interests previously held Laidlaw et al. (2004) highlight the importance of evaluating role investment when building a formulation
Intergenerational linkages	Research emphasises the vital role of grandparents and great‐grandparents in maintaining intergenerational connections (Bengston & Boss, 2000; Hünteler, 2022) Changes in family structure due to separation, divorce, and remarriage may cause tension and be misunderstood by older generations, potentially interacting with cohort beliefs
	Individuals may have various attitudes and beliefs about ageing, including internalised negative stereotypes (e.g. ‘growing old is an awful process’ or ‘I'm too old to change’ and assumptions about depression (e.g. this is a normal process of ageing))
Socio‐cultural context	Therapists should explore these beliefs during case formulation and when introducing therapy
Physical health	Growing older increases the likelihood of developing more physical health problems Laidlaw et al. (2004) emphasise the importance of inquiring about the presence and impact of any co‐morbid medical conditions

### Consultation with older adults

An important aspect of developing accessible, acceptable, and effective interventions is to ensure that it is done in collaboration with individuals who have lived experience (Michalak et al., 2016). Both Tyler et al. (2022) and Depp et al. (2007) reported that they sought input from older adults when creating their psychological interventions.

Depp et al. (2007) reported that during the development of the intervention, they received input from a Community Advisory Board (CAB), which included patient participants in research, family members, caregivers, and various professionals. The CAB provided feedback on the study design, particularly the structure and content of the group intervention. Based on the feedback from the CAB, the length of the intervention was set at 12 weeks and the duration of sessions was reduced to 1.5 hours to minimise participant fatigue.

Tyler et al. (2021) conducted a series of focus groups with older individuals with BD, contributing to the adaptation of the RfT intervention. Participants in the focus groups reported experiencing changes in their symptoms and the impact of physical and cognitive difficulties as they aged. This led to specific recommendations for adapting therapy for older individuals with BD.

Tyler et al.'s (2021) older adult BD specific adaptations include:

- Spend more time at the beginning of the therapy sessions to build a strong relationship and explore a longer and potentially more complex history.
- During the assessment phase, spend time with the older person to understand any changes in their symptoms of mania and depression over time and how they have coped with this.
- Recognise the impact of repeated episodes over time and the potential for an increased sense of hopelessness in making changes later in life.
- Explore the impact of having more time due to changes in role, and therefore more space to ruminate and experience feelings of guilt and shame related to past BD episodes.
- Help individuals build on areas of personal growth and enhance assertiveness and confidence to manage BD experiences, in line with recovery principles.
- Nurture existing strengths and acknowledge the resilience already present in living a life with a long‐term condition.

A detailed overview of all of the adaptations can be found in Tyler et al. (2021).

## FUTURE RESEARCH DIRECTIONS

This paper outlines that the literature surrounding psychological interventions for older adults with BD remains limited. The available research suggests that psychological interventions are valued by older adults with BD and have the potential to facilitate meaningful change. The four articles identified in this review highlighted that very different psychological approaches are often used to support older adults living with BD. These contrasting approaches are perhaps indicative of the varying and changing needs of individuals with BD in later life. Despite this, there remains no specific guidance from the National Institute of Health and Care Excellence (NICE, 2014) and no large‐scale randomised controlled trials investigating psychological approaches for older adults with BD.

In future, larger randomised controlled trials are necessary to build upon the findings of this review and determine the efficacy of psychological treatments for this group. Importantly, this paper identified that adaptations are both necessary and possible when offering psychological interventions for this population. Future research should continue to explore which adaptations are valued by older adults with BD. This could help to inform clinical practice by illustrating how services can tailor their services to meet the changing needs of this group. The importance of consulting older adults with BD when developing psychological interventions is also highlighted (Tyler et al., 2021). Warner et al. (2024) found that older adults with BD hold a wealth of knowledge about the challenges faced when living with BD in later life. Researchers should utilise this lived experience and collaborate alongside this population to ensure that future interventions are appropriate and effective for older adults with BD.

## CONCLUSIONS

At present, our knowledge about the psychological needs of older adults with BD is insufficient. This gap in our understanding is illuminated by the lack of available research investigating the efficacy of psychological treatments for this group. To address this gap, large‐scale clinical trials and increased investigation into how to support older adults with BD are warranted. Greater awareness of the challenges faced by older adults with BD can help to inform the development of appropriate treatments moving forward. This may help to reduce the significant inequalities this group currently faces and support them to live meaningful, fulfilling lives as they age.

## AUTHOR CONTRIBUTIONS

**Elizabeth Tyler:** Conceptualization; writing – review and editing; writing – original draft; methodology; investigation. **Aaron Warner:** Conceptualization; investigation; writing – original draft; methodology; writing – review and editing.

## CONFLICT OF INTEREST STATEMENT

The authors do not have any conflicts of interest.

## Data Availability

Data sharing is not applicable to this article as no new data were created or analyzed in this study.
